# *Drosophila* Immunity: Analysis of PGRP-SB1 Expression, Enzymatic Activity and Function

**DOI:** 10.1371/journal.pone.0017231

**Published:** 2011-02-18

**Authors:** Anna Zaidman-Rémy, Mickael Poidevin, Mireille Hervé, David P. Welchman, Juan C. Paredes, Carina Fahlander, Hakan Steiner, Dominique Mengin-Lecreulx, Bruno Lemaitre

**Affiliations:** 1 Centre de Génétique Moléculaire du CNRS, Gif-sur-Yvette, France; 2 Institut de Biochimie et Biophysique Moléculaire Cellulaire, UMR 8619 CNRS, Université Paris-Sud, Orsay, France; 3 EPFL, Global Health Institute, Lausanne, Switzerland; 4 Department of Genetics, Microbiology and Toxicology, Stockholm University, Stockholm, Sweden; Charité-University Medicine Berlin, Germany

## Abstract

Peptidoglycan is an essential and specific component of the bacterial cell wall and therefore is an ideal recognition signature for the immune system. Peptidoglycan recognition proteins (PGRPs) are conserved from insects to mammals and able to bind PGN (non-catalytic PGRPs) and, in some cases, to efficiently degrade it (catalytic PGRPs). In *Drosophila*, several non-catalytic PGRPs function as selective peptidoglycan receptors upstream of the Toll and Imd pathways, the two major signalling cascades regulating the systemic production of antimicrobial peptides. Recognition PGRPs specifically activate the Toll pathway in response to Lys-type peptidoglycan found in most Gram-positive bacteria and the Imd pathway in response to DAP-type peptidoglycan encountered in Gram-positive bacilli-type bacteria and in Gram-negative bacteria. Catalytic PGRPs on the other hand can potentially reduce the level of immune activation by scavenging peptidoglycan. In accordance with this, PGRP-LB and PGRP-SC1A/B/2 have been shown to act as negative regulators of the Imd pathway. In this study, we report a biochemical and genetic analysis of PGRP-SB1, a catalytic PGRP. Our data show that PGRP-SB1 is abundantly secreted into the hemolymph following Imd pathway activation in the fat body, and exhibits an enzymatic activity towards DAP-type polymeric peptidoglycan. We have generated a *PGRP-SB1/2* null mutant by homologous recombination, but its thorough phenotypic analysis did not reveal any immune function, suggesting a subtle role or redundancy of PGRP-SB1/2 with other molecules. Possible immune functions of PGRP-SB1 are discussed.

## Introduction

In the last decades, peptidoglycan (PGN), an essential component of virtually all bacteria, has appeared as a key player in host-microorganism interactions [1]. This glycopeptidic polymer is a component of the cell wall of both Gram-negative and Gram-positive bacteria. It consists of long glycan chains of alternating *N*-acetylglucosamine (GlcNAc) and *N*-acetylmuramic acid (MurNAc) residues, which are cross-linked together via short peptide bridges. Because PGN is essential to bacteria but absent from eukaryotic cells, it makes an ideal indicator for metazoan immune systems of the presence of bacteria within the organism. In addition, the PGN composition and structure can be markedly different between bacterial species, allowing the immune system to further distinguish between different types of intruders. For example, the PGN of Gram-negative and Gram-positive bacilli-type bacteria differs from the PGN of most Gram-positive bacteria by the replacement of lysine (Lys) with *meso*-diaminopimelic acid (DAP) at the third position in the peptide chain [2].

On the host side, some protein families have evolved the capacity to interact with PGN, e.g. lysozymes, Nods (for Nucleotide-binding Oligomerisation Domain), and peptidoglycan recognition proteins (PGRPs, or PGLYRPs in the mammalian nomenclature). PGRPs form a conserved family of proteins sharing a 160 amino acid domain (the PGRP domain) with similarities to bacteriophage T7 lysozyme, a zinc-dependent *N*-acetylmuramoyl-L-alanine amidase [3], [4]. First identified in *Bombyx mori* [5], PGRPs have since been intensively studied in several insects and vertebrates, including mammals [4], [6], [7]. These numerous studies have highlighted the diversity and importance of PGRP functions in immunity.

PGRPs have been classified in two groups according to their enzymatic activity. Indeed, some PGRPs have lost their ancestral amidase activity (non-catalytic PGRPs), while others can still efficiently cleave PGN (catalytic PGRPs). Non-catalytic PGRPs have been implicated in functions as diverse as immune receptors, regulators and effectors [4]. Catalytic PGRPs have been shown to down-regulate the immune response in insects, act as pro-inflammatory cytokines in mice, and have bactericidal activity in zebrafish [8], [9], [10], [11], [12]. Recently, it has been reported that mammalian PGRPs can prevent aberrant inflammatory responses by modulating the composition of the intestinal bacterial flora, a function in accordance with the strong expression of PGRPs along the digestive tract [12]. Moreover, catalytic PGRPs also participate in the establishment of symbiotic interactions in both squid and insects by preventing the activation of an immune response by their bacterial symbiont [13], [14], [15], [16].

The *Drosophila* genome encodes 13 PGRPs and many of them function both as activators and regulators of the Toll and Imd pathways [4], [17], [18]. These two major signalling cascades regulate the expression of antimicrobial peptides and other immune genes by the fat body after a systemic infection [19]. Activation of both pathways by bacteria relies on the sensing of specific forms of PGN by non-catalytic PGRPs [20]. PGRP-SA and PGRP-SD are secreted proteins circulating in the hemolymph that activate the Toll pathway in response to the Lys-type PGN of Gram-positive bacteria [21], [22]. PGRP-LC acts as a transmembrane receptor of Gram-negative and bacilli DAP-type PGN upstream of the Imd pathway, with the help of PGRP-LE [23], [24], [25], [26], [27]. The Imd pathway can be activated by both polymeric and monomeric DAP-type PGN, and the minimal PGN motif for its efficient induction is tracheal cytotoxin (TCT, or GlcNAc-MurNAc(anhydro)-L-Ala-γ-D-Glu-*meso*-DAP-D-Ala; [28], [29]). TCT provides an ideal indication of Gram-negative bacterial activity since this monomer, which is located at the terminus of PGN strands, is released during cell growth and division.

**Figure 1:**
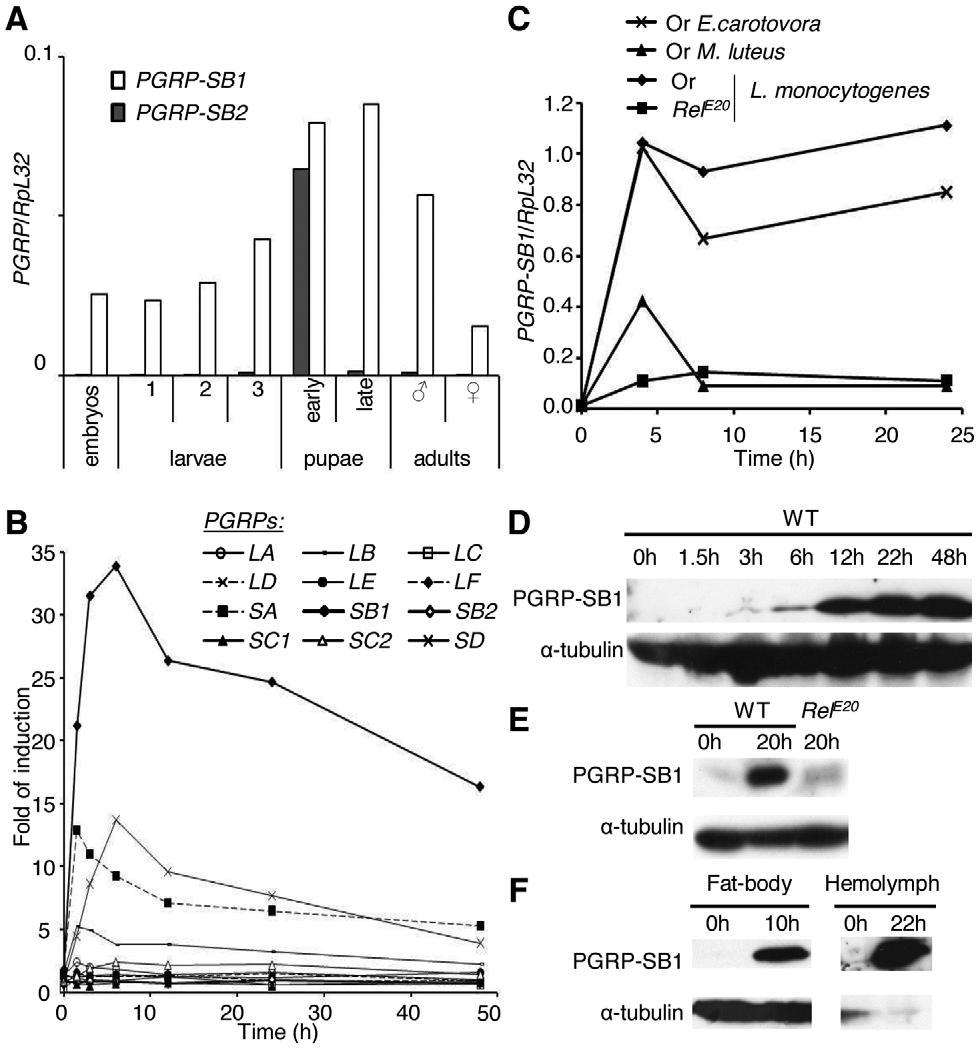
Expression profile of *PGRP-SB1*. (A) RT-qPCR measurements reveal a low basal expression of *PGRP-SB1* at all stages of the *Drosophila* life cycle, with a slight enhancement in pupae, while *PGRP-SB2* is solely expressed at pupal stage. (B) The expression profile of *PGRP-SB1* shows rapid induction after septic injury with a mixture of *E. coli* and *M. luteus*. This induction is much stronger than that observed for other *PGRPs*. The microarray data for this figure were extracted from [42]. (C) RT-qPCR analyses indicate that *PGRP-SB1* expression is induced upon immune challenge with Gram-negative bacteria *E. carotovora* or Gram-positive bacteria *L. monocytogenes*, both of which contain DAP-type PGN. *PGRP-SB1* is not induced upon *L. monocytogenes* infection in *Relish* flies deficient in Imd pathway activation. Septic injury with the Lys-type PGN containing Gram-positive bacteria *M. luteus* only weakly induced *PGRP-SB1* expression. Or: Oregon (wild-type); *Rel* ^E20^: *Relish* mutant flies. (D) The expression profile of PGRP-SB1 during the course of an infection is revealed by Western blot analysis. Proteins were extracted from male flies collected at different time points after infection. Wild-type (WT): Oregon. (E) PGRP-SB1 expression is induced upon immune challenge in wild-type flies but not in *Relish* flies. Wild-type (WT): Oregon; *Rel^E20^*: *Relish* mutant flies. (F) PGRP-SB1 expression is strongly induced in the fat body and the protein is secreted into the hemolymph. Western blot analyses were performed with proteins extracted from fat body or hemolymph derived from female flies collected at 10 h or 22 h post-infection, respectively. Hemolymph samples of 50 female flies were used to extract proteins, of which 15 µg were loaded (see Material and Methods). The absence of a signal with α-tubulin antibody indicates that the hemolymph preparations were not contaminated by cells.

While several *Drosophila* non-catalytic PGRPs function as pattern recognition receptors for PGN, the catalytic PGRPs have demonstrated (PGRP-SC1A/B, LB, SB1) or predicted (PGRP-SB2, SC2) amidase activity that removes peptides from PGN glycan chains, thereby reducing or eliminating its immunological activity [8], [30], [31], [32]. In accordance with this enzymatic activity, PGRP-SC1A/B/2 and PGRP-LB have been shown to modulate Imd pathway activation *in vivo* by scavenging PGN, and also TCT in the case of PGRP-LB [8], [9]. PGRP-SC1A has also been implicated in the phagocytosis of *Staphylococcus aureus* [33]. Among catalytic *Drosophila* PGRPs, only the function of *PGRP-SB1* and *PGRP-SB2* (which is only expressed at pupal stage; [34] - see Fig. 1A) had not yet been studied *in vivo*.

In this study, we provide a biochemical and genetic analysis of PGRP-SB1. Our data show that PGRP-SB1 is abundantly secreted into the hemolymph following Imd pathway activation in the fat body. Biochemical studies demonstrate that PGRP-SB1 has enzymatic activity towards DAP-type PGN but does not cleave TCT. Importantly, we generated a *PGRP-SB1/PGRP-SB2* double mutant by homologous recombination. We show that *PGRP-SB1/2* mutants are viable and do not display a striking phenotype in any of the classical immune parameters tested: resistance to infections, local and systemic activation of Toll and Imd pathways after septic or oral infections, and bacterial persistence in the fly.

## Results

### *PGRP-SB1* is strongly induced by the Imd pathway upon infection with bacteria containing DAP-type PGN

To characterize the function of *PGRP-SB1*, we first analyzed its expression pattern. A Real Time quantitative Polymerase Chain Reaction (RT-qPCR) analysis revealed a basal level of *PGRP-SB1* expression throughout *Drosophila* development; however, its expression level was higher at pupal stage, consistent with a previous Northern blot analysis (Fig. 1A; [34]). Previous microarray analysis performed in adult males indicated that *PGRP-SB1* is induced 35-fold following septic injury with a mixture of *Micrococcus luteus* and *Escherichia coli* [35]. The comparison of the different expression profiles revealed that *PGRP-SB1* was by far the strongest induced *PGRP* after bacterial infection (Fig. 1B). To investigate whether *PGRP-SB1* expression was equally induced by different bacterial challenges, we monitored *PGRP-SB1* expression after injection of the Gram-negative bacteria *Erwinia carotovora*, Gram-positive *Listeria monocytogenes* or Gram-positive *M. luteus*. *PGRP-SB1* expression was strongly induced 4 h after infection with *E. carotovora* or *L. monocytogenes*, both of which contain DAP-type PGN, and remained sustained during the course of infection (Fig. 1C). In contrast, flies infected with *M. luteus*, which possesses Lys-type PGN, exhibited only a slight and brief induction of *PGRP-SB1*. In agreement with the microarray analysis [35], we confirmed that the induction of *PGRP-SB1* expression after *E. carotovora* or *L. monocytogenes* injection did not occur in *Relish* mutant flies lacking a functional Imd pathway (Fig. 1C).

We next analyzed the expression profile of PGRP-SB1 protein using a mouse serum raised against PGRP-LB [8] that cross-reacts with PGRP-SB1 (data not shown). Western blots indicated that PGRP-SB1 expression was weak in unchallenged adult males, while the expression level increased 6 h after septic injury with *E. carotovora* and reached a maximum at 22 h after infection (Fig. 1D). PGRP-SB1 was detected as a single band of 18 kDa as predicted by the genome annotation. In agreement with the mRNA analysis, PGRP-SB1 was not induced after injection of *E. carotovora* in *Relish* mutant flies (Fig. 1E). PGRP-SB1 was abundant in protein extracts from both the hemolymph and the fat body (Fig. 1F). This indicates that PGRP-SB1 is produced in the fat body upon systemic infection and secreted into the hemolymph, in agreement with the presence of a signal peptide in the protein.

Altogether, these results indicate that stimulation of the Imd pathway by bacteria containing DAP-type PGN leads to rapid PGRP-SB1 synthesis and secretion into the hemolymph.

### PGRP-SB1 cleaves DAP-type PGN and specific PGN fragments, but does not degrade TCT

**Figure 2:**
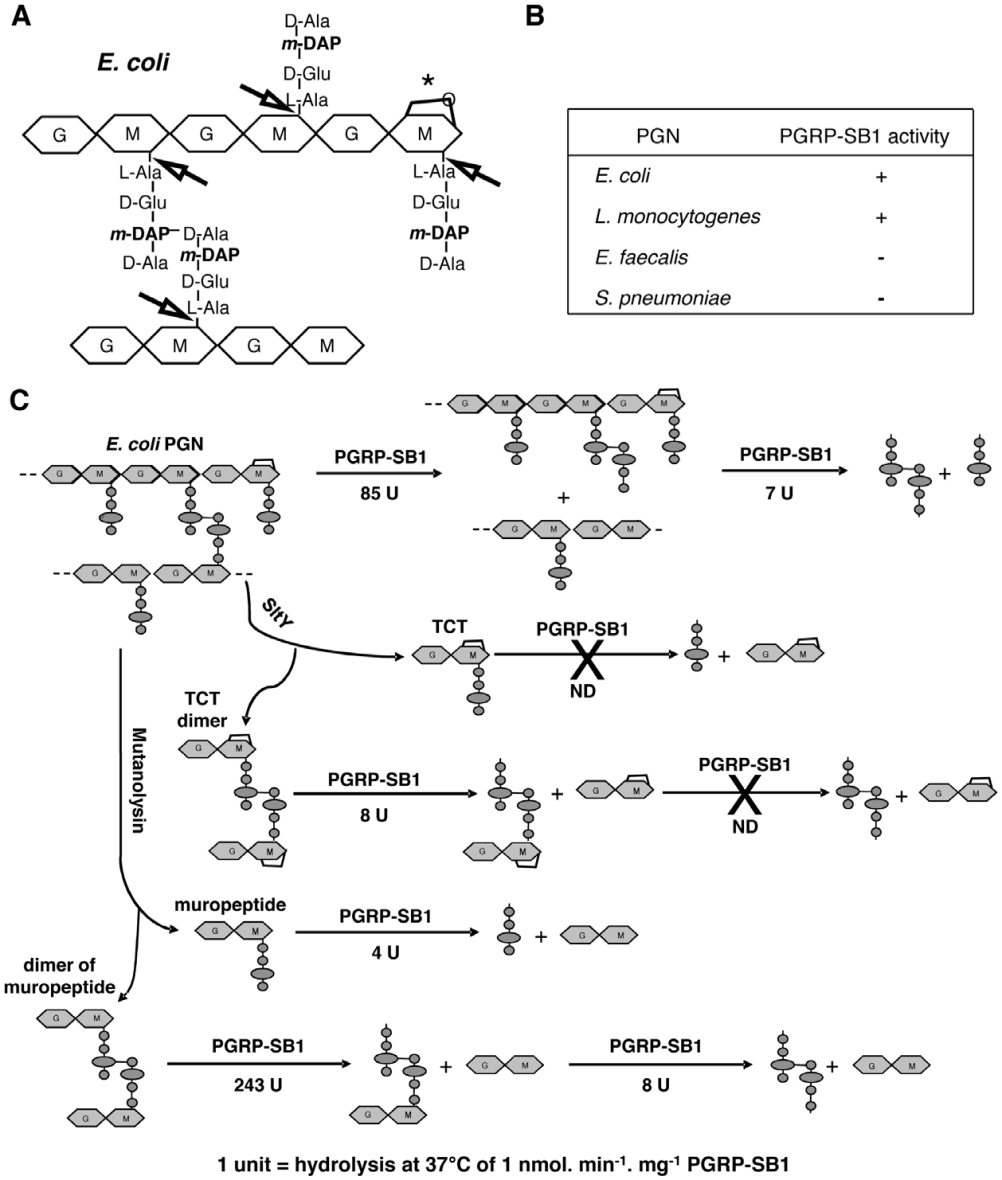
Amidase activity and substrate specificity of recombinant PGRP-SB1. (A) Structure of *E. coli* PGN (DAP-type). The glycan chains are formed of alternating GlcNAc (G) and MurNAc (M) residues. Those of *E. coli* PGN end with a 1,6-anhydro-MurNAc residue (*). The tetrapeptide motif corresponds to the TCT. Arrows indicate the cleavage sites of *N*-acetylmuramoyl-L-alanine amidases. The main peptides released following treatment of cross-linked PGN with PGRP-SB1 are the tetrapeptide L-Ala-γ-D-Glu-*meso*-DAP-D-Ala and its dimer, the octapeptide. (B) Specificity of PGRP-SB1 on Lys-type and DAP-type PGN was determined by measuring its amidase activity on PGN derived from four bacterial species: *E. coli*, *L. monocytogenes* (DAP-type PGN) and *E. faecalis* and *S. pneumoniae* (Lys-type PGN). The amidase activity was followed by analyzing by HPLC the release of tri- and tetrapeptides and of the corresponding dimers in solution, incubating for 26 h 100 µg of PGN with 2 µg of PGRP-SB1. (C) The PGRP-SB1 activity was determined on *E. coli* polymeric PGN and various purified PGN fragments. These fragments were obtained after treatment of *E. coli* PGN either with mutanolysin or SltY, generating monomers and dimers of muropeptides (GlcNAc-MurNAc-tetrapeptide units) or anhydro-muropeptides (GlcNAc-MurNAc(anhydro)-tetrapeptide units, i.e. TCT), respectively. Substrates and products were separated by HPLC, characterized and quantified. The results are summarized in the scheme and the PGRP-SB1 activity is given in units, 1 unit representing 1 nmol of substrate hydrolyzed per min and per mg of PGRP-SB1.

PGRP-SB1 has been shown to have an amidase activity towards DAP-type PGN [32]. PGRP-SB1-mediated PGN cleavage was much weaker towards *Lactobacillus casei* Lys-type PGN and no activity was detected towards *S. aureus* and *M. luteus* Lys-type PGN [32]. We extended this analysis by identifying the minimum PGN motif recognized by PGRP-SB1. In agreement with Mellroth and Steiner, incubating purified DAP-type PGN derived from *E. coli* and *L. monocytogenes* (see structure in Fig. 2A) with PGRP-SB1 at 37°C resulted in the release of soluble tri-, tetra-, tri-tetra- and tetra-tetra-peptides, indicative of an *N*-acetyl muramoyl-L-alanine amidase activity. Such an activity was not detected against Lys-type PGN derived from *Enterococcus faecalis* or *Streptococcus pneumoniae* (Fig. 2B, 2C).

TCT (GlcNAc-MurNAc(anhydro)-L-Ala-γ-D-Glu-*meso*-DAP-D-Ala) has been previously identified as the minimum PGN motif capable of efficiently inducing the Imd pathway [28], [29]. No amidase activity was observed following incubation at 37°C for 16 h of 2 µg of PGRP-SB1 with 20 nmol of TCT (Fig. 2C) contrary to what has been shown with PGRP-LB [8]. However, incubating PGRP-SB1 in the same conditions with TCT dimer resulted in the cleavage at only one of the two putative amidase sites, generating disaccharide-octapeptide and free dissacharide (Fig. 2C). The same experiment with PGRP-LB resulted in the production of octapeptide and disaccharide [8]. We next analyzed the effect of PGRP-SB1 on monomer and dimer (with no anhydro bond) that were purified from mutanolysin treated *E. coli* DAP-type PGN. Both the monomer (GlcNAc-MurNAc tetrapeptide unit) and dimer of muropeptide were hydrolyzed by PGRP-SB1 but PGRP-SB1 was much more active on the dimer (243U compared to 4U for the monomer). Kinetic studies of the hydrolysis of GlcNAc-MurNAc tetrapeptide dimer showed a two step-reaction, the faster one leading to disaccharide-octapeptide and disaccharide and the slower one leading to octapeptide and disaccharide (Fig. 2C). This prompted us to test whether such a preference of PGRP-SB1 for dimeric structures was similarly observed when polymeric PGN was used as substrate. *E. coli* PGN was treated by PGRP-SB1 for different times and the resulting modifications of the polymer structure were analyzed by a classical procedure based on HPLC analysis of the pattern of muropeptides released after degradation of PGN material by SltY. It was observed that PGRP-SB1 indeed also hydrolyzed dimeric structures present in PGN much faster (at least 10-fold) than monomeric ones (Fig. 2C).

Collectively, our results demonstrate that PGRP-SB1 degrades DAP-type PGN into non-immunostimulatory fragments, but does not degrade TCT, a key activator of the Imd pathway. We also observed that the efficacy of PGRP-SB1 is dependent on two factors, the size of the PGN fragments (monomer, dimer or polymer) and the MurNAc configuration (anhydro or not).

### Generation of a *PGRP-SB1*/*PGRP-SB2* mutant by homologous recombination

**Figure 3:**
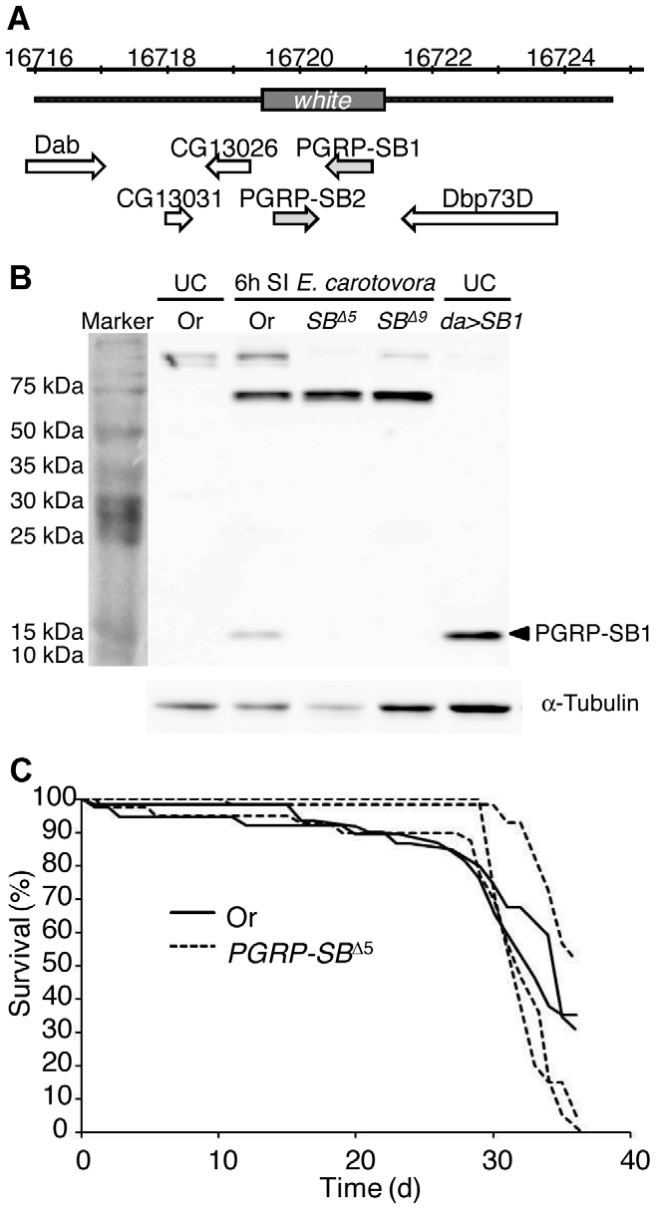
Generation of a null *PGRP-SB1/2* mutation by homologous recombination. (A) Schematic representation of the *PGRP-SB1/2* deletion. The gene map was adapted from FlyBase (http://flybase.org/) and includes *PGRP-SB1*, *PGRP-SB2* and the neighboring genes. The deleted segment replaced by the *white* gene (grey box) and the flanking sequences used for recombination (dotted lines) are indicated. (B) PGRP-SB1 is not expressed in *PGRP-SB* ^Δ5^ and *PGRP-SB* ^Δ9^ mutant flies. The PGRP-SB1 protein cross-reacts with a serum raised against PGRP-LB and appeared as a band of about 18 kDa on this Western blot (arrow head). The band was detected in wild-type female extracts (Or: Oregon) 6 h after injection of *E. Carotovora*. The protein was also detected in *daughterless-Gal4/UAS-PGRP-SB1* (*da>SB1*) flies over-expressing PGRP-SB1 in unchallenged condition. In contrast, the protein was not detected in uninfected wild-type flies or in *PGRP-SB* ^Δ5^ (*SB* ^Δ5^) and *PGRP-SB* ^Δ9^ (*SB* ^Δ9^) mutants 6 h after injection of *E. Carotovora*. SI: septic injury; UC: Unchallenged. (C) Lifespan is not affected in *PGRP-SB* ^Δ5^ mutant flies. The survival rates (%) of WT and *PGRP-SB* ^Δ5^ mutant flies at 29°C were measured over 36 days and did not reveal any differences in longevity. Each line represents an independent experiment for that genotype and gives the mean survival of 2-3 cohorts of 20 flies within that experiment.

In order to address the function of *PGRP-SB1 in vivo*, we aimed to generate a null mutant by homologous recombination. The *PGRP-SB1* locus (720 bp) is located on the 3L chromosome at less than 150 bp from *PGRP-SB2* (615 bp; Fig. 3A). The two corresponding proteins, of 190 and 182 amino acids respectively, share 68% similarity (51% identity), but the genes differ in their expression, as *PGRP-SB2* is mainly expressed at the pupal stage (Fig. 1A and [34]). We used a homologous recombination approach to replace approximately 1.5 kb, corresponding to the ORFs of the two genes, with a copy of the *white* gene (Fig. 3A). We obtained several independent fly lines and two of them, *PGRP-SB* ^Δ5^ and *PGRP-SB* ^Δ9^, were selected for further analysis. We used RT-qPCR and Western blot to confirm the absence of *PGRP-SB2* transcript and PGRP-SB1 protein respectively in homozygous *PGRP-SB* ^Δ5^ and *PGRP-SB* ^Δ9^ mutants (Fig. 3B and data not shown). We checked that the *PGRP-SB1/2* deletion did not affect the expression of the flanking genes *CG13026* and *Dbp73D* (Fig. 3A and data not shown). As both alleles produced identical results, we only report here data obtained with *PGRP-SB* ^Δ5^. These mutants were perfectly viable and fertile, exhibited no visible developmental defects and did not differ from wild-type flies in longevity (Fig. 3C).

### Analysis of the immune phenotype of *PGRP-SB* ^Δ5^ mutants

#### *PGRP-SB* ^Δ5^ mutants exhibit a wild-type resistance to systemic microbial infections

**Figure 4:**
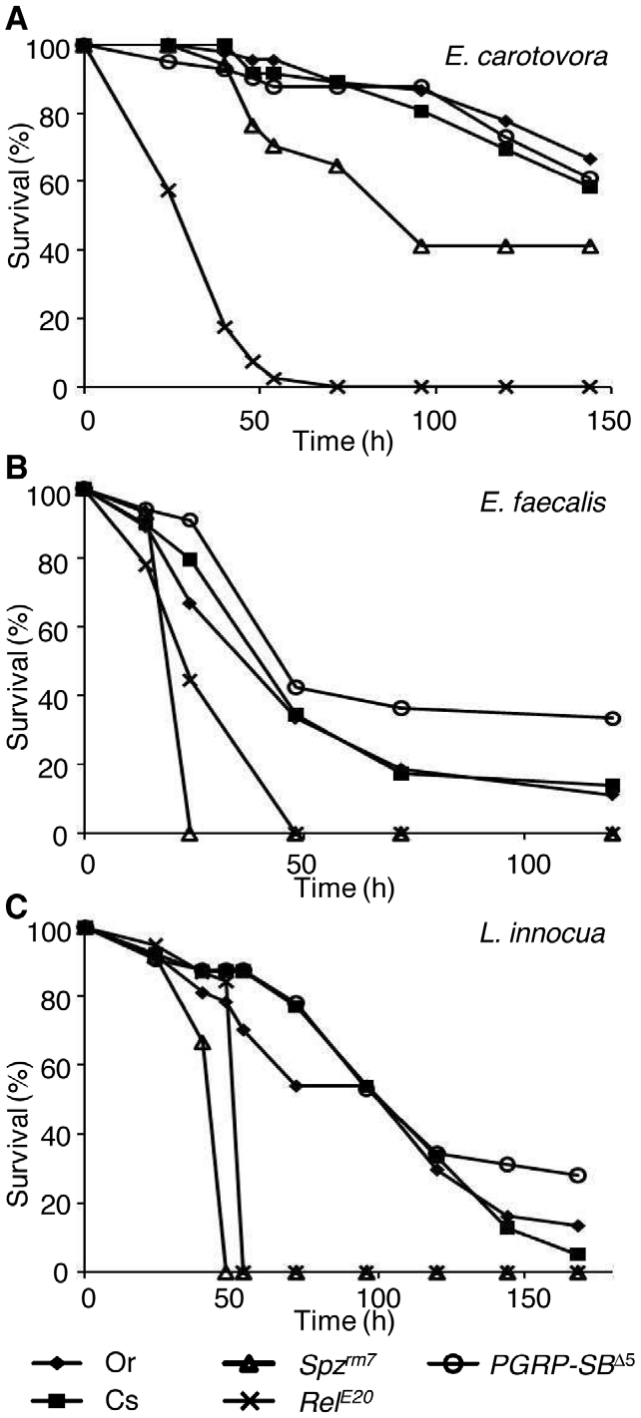
*PGRP-SB1* is not required for resistance to bacterial infections. The survival rates (%) of *PGRP-SB* ^Δ5^ mutant flies injected with the DAP-type PGN containing Gram-negative bacteria *E. carotovora* (A), the Lys-type PGN containing Gram-positive bacteria *E. faecalis* (B) or the DAP-type PGN containing Gram-positive bacteria *L. innocua* (C) were compared to wild-type flies Oregon (Or) and Canton-S (Cs) and flies defective in either the Toll pathway (*spätzle* mutants; *spz^rm7^*) or the Imd pathway (*Relish* mutants; *Rel^E20^*). *PGRP-SB* ^Δ5^ mutation did not modify the fly resistance to these bacterial infections. Optical densities of the bacterial strains used in these experiments are indicated in the Material and Methods section. One representative experiment out of three (*E. carotovora*, *L. innocua*) or two (*E. faecalis*) repeats is shown.

To determine the function of *PGRP-SB1* in the *Drosophila* immune response, we assayed the susceptibility of *PGRP-SB* ^Δ5^ mutants to injection of different classes of bacteria. *PGRP-SB* ^Δ5^ mutants were as resistant as wild-type flies to infections by DAP-type Gram-negative bacteria *E. carotovora*, DAP-type Gram-positive bacteria *L. innocua* and Lys-type Gram-positive bacteria *E. faecalis* (Fig. 4).

#### Induction of the systemic immune response after injection of bacteria or PGN is not affected in *PGRP-SB* ^Δ5^ mutants

**Figure 5:**
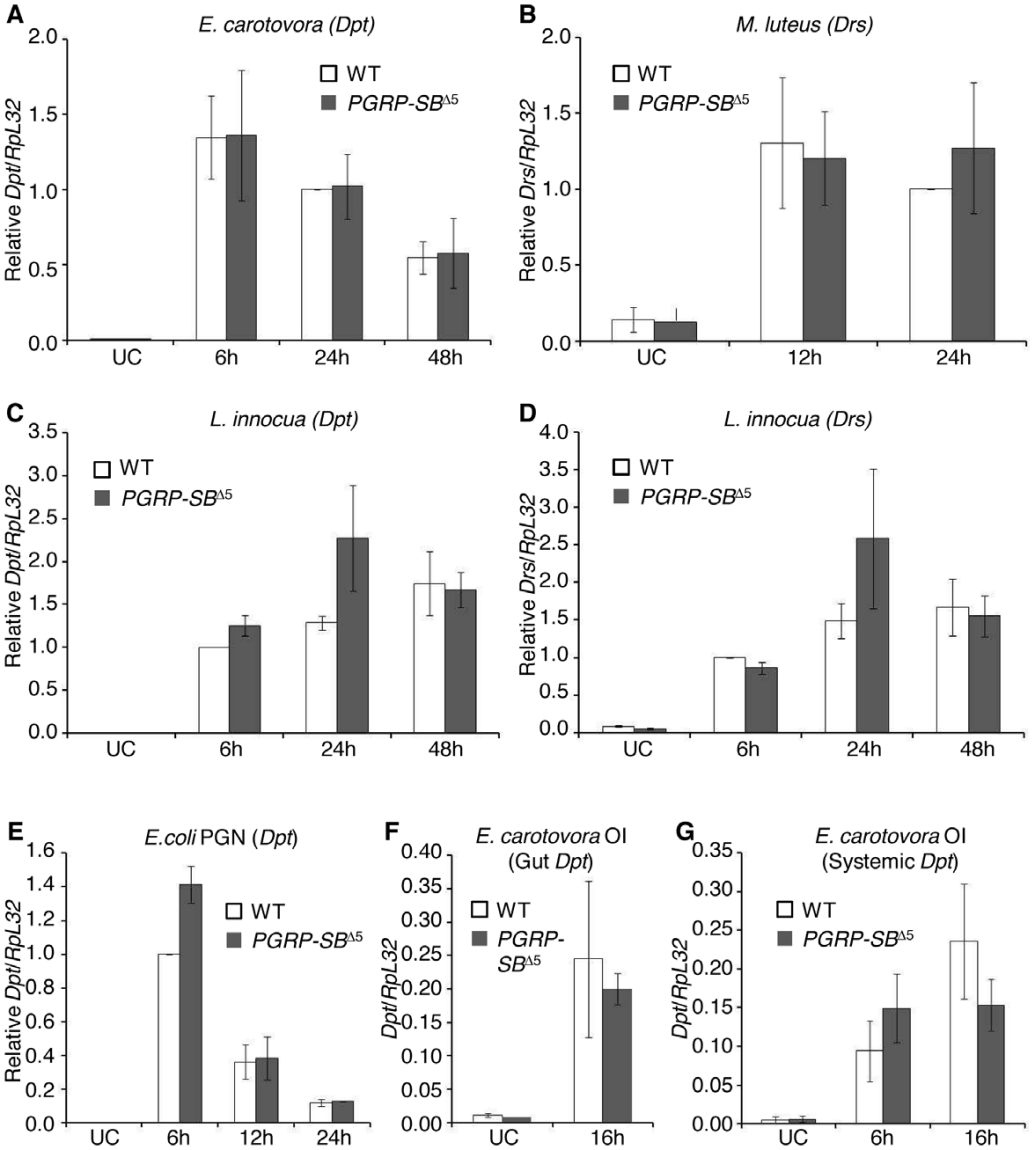
Antimicrobial peptide gene expressions in *PGRP-SB1/2* mutant flies after different immune challenges. (A) Injection of *E. carotovora* in *PGRP-SB* ^Δ5^ mutants induced *Diptericin* (*Dpt*) expression, a read-out of the Imd pathway activation, at a similar level to wild-type flies. (B) *PGRP-SB* ^Δ5^ mutants injected with *M. luteus* expressed *Drosomycin* (*Drs*), a read-out of the Toll pathway activation, at a wild-type level. (C) Injection of *L. innocua* in *PGRP-SB* ^Δ5^ mutants induced *Dpt* expression at a similar level to wild-type flies, except at 24 h after infection when *Dpt* expression was slightly higher in *PGRP-SB* ^Δ5^ mutants than in wild-type flies. (D) Injection of *L. innocua* in *PGRP-SB* ^Δ5^ mutants induced *Drs* expression at a similar level to wild-type flies. (E) Injection of *E. coli PGN* in *PGRP-SB* ^Δ5^ mutants induced *Dpt* expression at a similar level to wild-type flies, except at 6 h after injection when *Dpt* expression was slightly higher in *PGRP-SB* ^Δ5^ mutants than in wild-type flies. (F and G) *PGRP-SB* ^Δ5^ mutants orally infected (OI) with *E. carotovora* expressed *Dpt* at a wild-type level both locally in the gut (F) and systemically in the fat body (G). UC: unchallenged. *Dpt* or *Drs* gene expression was monitored by RT-qPCR performed on total RNA extracts from wild-type (WT; Oregon) and *PGRP-SB* ^Δ5^ mutant females. mRNA levels were normalized to *RpL32* mRNA. (A–E) The ratios indicated are either relative to wild-type ratio at 24 h (A and B) or 6 h (C–E) after infection, or absolute (F–G). The results are presented as the mean and standard error of three independent experiments.

We next tested the ability of *PGRP-SB* ^Δ5^ mutants to mount an efficient but regulated immune response after injection of various bacteria. Other members of the PGRP family have been implicated in both activation of the Toll and Imd pathways (PGRP-SA, PGRP-LC, PGRP-LE and PGRP-SD) and down-regulation of the Imd pathway (PGRP-LF, PGRP-LB and PGRP-SC1A/B/2) [4]. We monitored Toll and Imd pathway activation after different types of immune challenge by measuring the expression of two of their target genes, *Drosomycin* (*Drs*) and *Diptericin* (*Dpt*) respectively. *PGRP-SB* ^Δ5^ mutants expressed *Drs* and *Dpt* at a wild-type level after injection of *E. carotovora* (Fig. 5A), *M. luteus* (Fig. 5B), *L. innocua* (Fig. 5C and 5D), or *E. coli* PGN (Fig. 5E). However, *Dpt* expression was slightly higher in *PGRP-SB* ^Δ5^ mutants 24 h after infection with *L. innocua* and 6 h after injection of *E. coli* PGN (Fig. 5C and 5E). The mild over-activation of the Imd pathway at one time point after these two challenges could indicate a moderate action of PGRP-SB1 as a negative regulator, likely to be masked in most of the cases by the action of more efficient regulators such as PGRP-LB.

#### Neither local nor systemic activation of the Imd pathway after *E. carotovora* oral infection is affected by the loss of *PGRP-SB1/2*

*PGRP-SB1* is strongly induced in the fly gut after oral infection with Gram-negative bacteria ([36] and data not shown), suggesting a role in the intestinal immune response. We evaluated such a function by measuring the level of induction of the Imd pathway both locally and systemically after *E. carotovora* oral infection. In wild-type adult flies, *E. carotovora* ingestion induces a local immune response but barely any systemic immune response in the fat body. In contrast to *PGRP-LB RNAi* flies [8], *PGRP-SB* ^Δ5^ mutants did not present an increased local immune response or a strong activation of the systemic response as compared to wild-type flies (Fig. 5F and 5G). We conclude that *PGRP-SB1/2* do not play a major role in the regulation of antibacterial peptide genes after oral infection of adult flies.

#### *E. carotovora* and *L. innocua* persistence is similar in *PGRP-SB^Δ5^* mutants and wild-type flies

**Figure 6:**
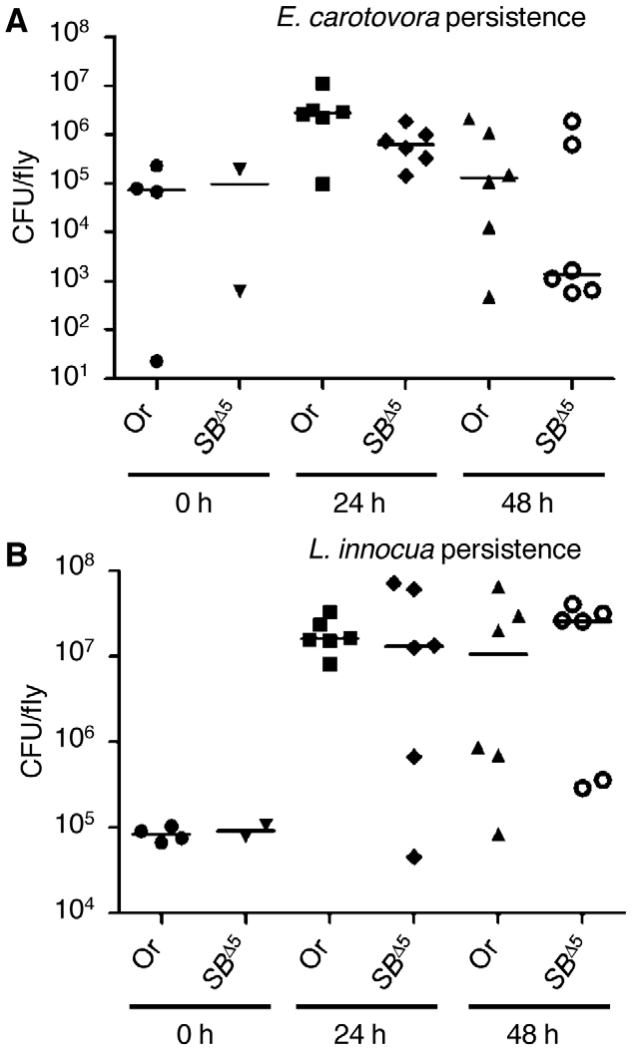
Persistence of *E. carotovora* and *L. innocua* bacteria is not affected by the *PGRP-SB* ^Δ5^ mutation. Persistence of *E. carotovora* (A) and *L. innocua* (B) bacteria in *PGRP-SB* ^Δ5^ mutant flies and wild-type flies was assayed at 0 h, 24 h and 48 h after injection, by spotting serial dilutions of fly extracts on LB- or BHI-agar Petri dishes (20 flies per data point). The values, which correspond to the results of three independent experiments, each made in duplicate, indicate the number of CFU (colony-forming unit) per fly. The median is indicated by a horizontal line. Wild-type flies: Oregon (Or); *PGRP-SB* ^Δ5^ mutant flies: *SB* ^Δ5^.

Our data indicate that, in contrast to all *PGRPs* studied so far, the lack of *PGRP-SB1/2* does not affect either Toll or Imd pathway activity. Strikingly, *PGRP-SB1* is the most highly inducible *PGRP*, with an induction level similar to that observed for antimicrobial peptide genes. A previous study has indicated that PGRP-SB1 displays some antibacterial activity against *B. megaterium in vitro* [32]. This suggests that PGRP-SB1 could be an effector molecule, participating in microbe elimination. To test this hypothesis we monitored the persistence of *E. carotovora*, *L. innocua* and *B. megaterium* 24 h and 48 h after their injection into wild-type or *PGRP-SB* ^Δ5^ mutant flies. Whole fly extracts were plated on LB or BHI-agar medium to assess the number of recovered bacterial colonies at different time points after infection. Our data indicated that *E. carotovora* (Fig. 6A) and *L. innocua* (Fig. 6B) did not persist better in *PGRP-SB* ^Δ5^ mutants than in wild-type flies, although surprisingly, the median persistence of *E. carotovora* at 48 h was somewhat lower in *PGRP-SB* ^Δ5^ mutants than in wild-type flies. *B. megaterium* appeared unable to survive injection into flies, preventing an assessment of the effect of *PGRP-SB1/2* mutations on its persistence (data not shown).

### PGRP-SB1 over-expression is not sufficient to protect from *L.innocua* infection

**Figure 7:**
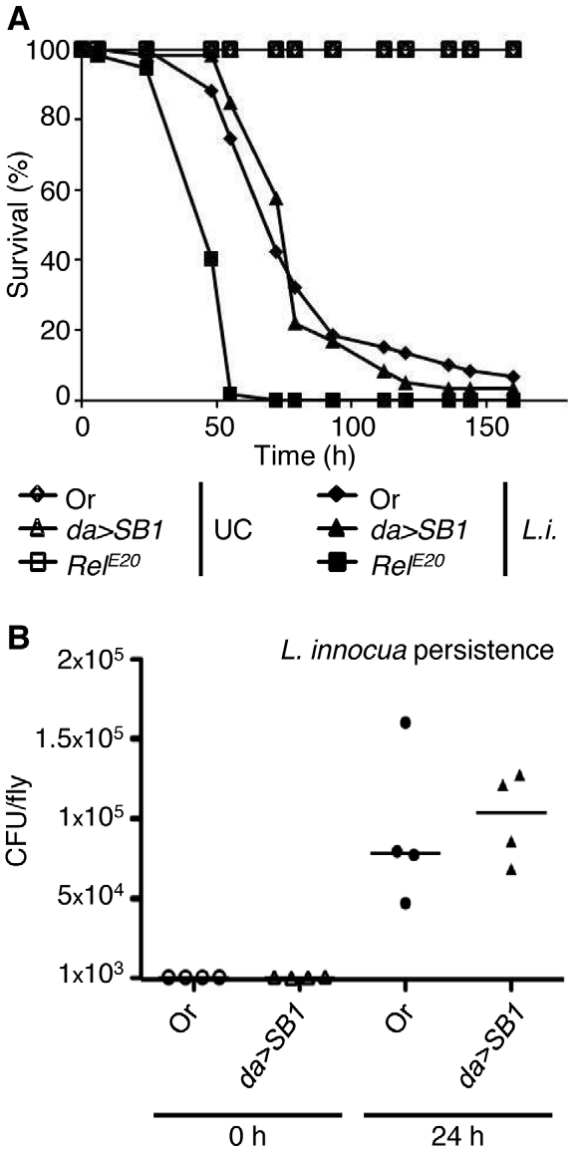
Over-expression of PGRP-SB1 is not sufficient to protect from *L. innocua* infection. (A) The survival rate (%) of flies over-expressing PGRP-SB1 (*daughterless(da)-Gal4/UAS-PGRP-SB1; da>SB1*) injected with the DAP-type PGN containing Gram-positive bacteria *L. innocua* was compared to the survival rate of wild-type flies Oregon (Or) and flies defective in the Imd pathway (*Relish* mutants; *Rel^E20^*). *PGRP-SB1* over-expression did not modify the fly resistance to this infection. *L.i.: L. innocua*; UC: unchallenged. One representative experiment out of three is shown. (B) Persistence of *L. innocua* bacteria in flies over-expressing PGRP-SB1 (*da>SB1*) and wild-type flies (Oregon; Or) was assayed at 0 h and 24 h after injection, by spotting serial dilutions of fly extracts on LB- or BHI-agar Petri dishes (20 flies per data point). The values, which correspond to the results of two independent experiments, each made in duplicate, indicate the number of CFU (colony-forming unit) per fly. The median is indicated by a horizontal line.

PGRP-SB1 being an efficient enzyme for DAP-type PGN degradation, we last aimed to test the hypothesis that PGRP-SB1 had a protective function against Gram-positive DAP-type containing bacteria, such as *Listeria*. Indeed, in these bacteria the PGN participates to the outer cell wall layer and could thus be directly accessible for PGRP-SB1, while in Gram-negative bacteria the PGN is “hidden” by the outer lipopolysaccharide layer. Therefore, we injected *L. innocua* in flies over-expressing PGRP-SB1 and assessed their viability as well as the bacteria persistence in these flies as compared to wild-type flies. Our data show that PGRP-SB1 over-expression is not sufficient to protect from the morbidity associated with this infection, or to decrease the number of persisting bacteria in the fly at 24 hours (Fig. 7).

In conclusion, our thorough analysis of various immune parameters (resistance to infections, activation of local and systemic immune responses after infection by injection or feeding, bacterial persistence in the fly) did not reveal a striking immune function for *PGRP-SB1* and *SB2*, despite the use of a null mutant.

## Discussion

The *PGRP* family has been thoroughly studied in the last decade, both in *Drosophila* and in vertebrates. We present here an extensive analysis of the expression, enzymatic activity and immune phenotype of *PGRP-SB1*, one of the last *Drosophila PGRPs* (alongside *PGRP-LA* and *PGRP-LD*) whose functions remain uncharacterized *in vivo*. This is also the first analysis of a complete genetic knockout of two catalytic PGRPs in *Drosophila*, as previously published *in vivo* studies of *PGRP-LB* and *PGRP-SC1/2* have relied either on RNA interference or on uncharacterized mutations [8], [9], [33].

In this study, we have demonstrated that *PGRP-SB1* expression is highly induced after infection, far more than for any other PGRP and to an extent similar to that of effectors such as antimicrobial peptide genes. PGRP-SB1 is abundantly produced in the fat body, and then released into the hemolymph. It has an amidase activity against DAP-type PGN. Taken together with the *in vitro* bactericidal activity of PGRP-SB1 against a DAP-type containing bacteria [32] these data suggest an effector function. Although our mutant analysis did not reveal any increase in susceptibility to infection or in bacterial persistence in *PGRP-SB* ^Δ5^ flies, it is expected that the removal of one out of many effectors would not induce a major immune deficiency, due to redundancy or synergy. This could also explain the non-protective effect of PGRP-SB1 over-expression after *L. innocua* infection. For example, PGRP-SB1 could need the activity of another protein to have sufficient access to its PGN substrate. On the other hand, PGN cleavage by PGRP-SB1 could increase with its over-expression but not be sufficient to eliminate the bacteria in absence of expression of a co-factor. Therefore a function of PGRP-SB1 as an effector remains a possibility.

There are several other possible explanations for the lack of an immune phenotype in the *PGRP-SB* ^Δ5^ mutants. Firstly, *PGRP-SB1/2* could have a function at a specific stage or tissue that would not be revealed by our analysis, based as it was on whole organism infections/responses and restricted to the adult stage. Indeed, *PGRP-SB2* is solely expressed at the pupal stage, suggesting that it could have a role during metamorphosis, although we did not notice any developmental defect of *PGRP-SB* ^Δ5^ mutants. Secondly, *PGRP-SB1* could have a more subtle immune function against a specific natural pathogen of the fly, untested here, or in other immune reactions, e.g. phagocytosis (as published for PGRP-SC1A [33]). Thirdly, as the deletion we have generated affects both *PGRP-SB1* and *SB2*, we cannot exclude the possibility that the phenotype of one mutation is suppressed by the phenotype of the other, as could be the case if the functions of these two genes were antagonistic. However, we consider this hypothesis unlikely because of the restriction of *PGRP-SB2* expression to the pupal stage. Finally, *PGRP-SB1* could have redundant regulatory functions with other catalytic PGRPs, as suggested by the mild and time restricted over-activation of the Imd pathway we noticed after injection of *L. innocua* bacteria and *E. coli* PGN. This hypothesis could be addressed by generating fly lines carrying mutations of multiple *PGRPs*.

Our data demonstrate that while it efficiently degrades polymeric and DAP-type PGN fragments, PGRP-SB1, unlike PGRP-LB, does not cleave TCT ([8] and this study). This suggests that the presence of large quantities of PGRP-SB1 in the hemolymph, in spite of degrading PGN polymers, would not fully suppress Imd pathway activation by Gram-negative bacteria, due to the fact that these bacteria constantly release TCT during growth and division. By contrast, PGRP-LB degrades all immunogenic forms of DAP-type PGN and thus can completely suppress the immune response to PGN [8]. This substrate specificity among catalytic PGRPs might reflect a requirement for an effector function which does not eliminate the immune response. The specificity of PGRP-SB1 enzymatic activity could contribute to the elimination of bacteria by degrading their cell wall, while allowing TCT to accumulate and continue to activate the other branches of the immune response. Similarly, the cleavage of PGN could allow a distinction to be made between dead bacteria, whose lysis would release polymeric PGN, and alive, rapidly dividing bacteria, which are potentially harmful and would release TCT. The substrate specificity of PGRPs might thus allow fine-tuning of the immune response. From this point of view, it would be interesting to determine the substrate specificity and minimal molecular requirements of PGRP-SC1A, SC1B and SC2.

Recent work on PGRPs has highlighted the complexity of the interactions between bacterial PGN and host proteins [37]. In addition, it seems that these interactions have evolved differently depending on needs and bacterial exposure of the host species. Thanks to the broad spectrum of their ligand and substrate specificities, PGRPs function both as symbiosis facilitators and as immune activators, effectors and regulators [4], [13], [14], [15], [16], [18]. This diversification reflects the importance of PGN/host protein interactions for the communication between symbiotic, commensal or pathogenic bacteria and their host. We believe that the systematic analysis of PGRP expression patterns, enzymatic activities and *in vivo* functions is a powerful approach to decipher the complexity of these interactions.

## Materials and Methods

### Fly stocks and transgenic and mutant generation

Oregon^R^ flies were used as wild-type controls. *spz^rm7^* and *Rel^E20^* alleles are null mutations in *spätzle* and *Relish* respectively [20], [38]. *Drosophila* stocks were maintained at 25°C using standard fly medium.

A deletion of the *PGRP-SB1* and *SB2* locus was obtained by homologous recombination [39]. 3,715 bp and 4,252 bp sequences of DNA flanking the 5′ and 3′ ends, respectively, of the *PGRP-SB1/2* locus (Fig. 3A) were cloned in the pW25 vector [39]. The primers used were F: 5′-GCAGCGGCCGC CAGTTGCAATTCCACGCC-3′ and R: 5′-CGTGGTACCCTTTCGGTCACCGATCTGC-3′ for the 3,715 bp fragment, and F: 5′-GCAGGCGCGCCTTTTACGGGAAACGAAGCG-3′ and R: 5′-CGTCGTACGAATCCGGCACATGTGCG-3′ for the 4,252 bp fragment. 1,525 bp of *PGRP-SB1* and *SB2* sequences (3L: from nucleotide 16719625 to 16721150) were replaced by the *white* gene.

To generate *UAS-PGRP-SB1* transgenic flies, a full-length cDNA of *PGRP-SB1* (using the *CG9681*_cDNA full length IP02762 from DGRC) was inserted in the pDONR221 Gateway entry clone (Invitrogen) and finally subcloned in pTW transgenesis vector. The transgenic flies were obtained by P-element transgenesis. Over-expression of *PGRP-SB1* was achieved by crossing the resulting *UAS-PGRP-SB1* transgenic flies with flies carrying the ubiquitous *daughterless-Gal4* driver. F1 progeny was transferred to 29°C at late pupal stage for optimal efficiency of the UAS/Gal4 system.

### Bacterial strains and infection experiments

Systemic bacterial infections were performed by pricking adults in the thorax with a thin needle previously dipped into a concentrated pellet of a bacterial culture [40]. Infected flies were subsequently maintained at 29°C. A minimum of 40 flies were used for survival experiments and survival was scored either twice a day or daily as appropriate. The bacteria strains used and their respective optical density (O.D.) at 590 nm were: the DAP-type PGN containing Gram-negative bacteria *Erwinia carotovora* [41] (*E. carotovora*, O.D. 200), the DAP-type PGN containing Gram-positive bacteria *Listeria monocytogenes* (*L. monocytogenes*, O.D. 80), *Listeria innocua* (*L. innocua*, O.D. 200) or *Bacillus megaterium* (*B. Megaterium*, O.D. 200), and the Lys-type PGN containing Gram-positive bacteria *Micrococcus luteus* (*M. luteus*, O.D. 200) and *Enterococcus faecalis* (*E. faecalis*, O.D. 15). Strains were cultured in Brain-Heart Infusion Broth (BHI - *Listeria*) or Luria Broth (LB - all others) at 29°C (*E. carotovora*) or 37°C (*Listeria, E. faecalis, B. megaterium, M. luteus*). *E. coli* PGN was used at a monomer equivalent concentration of 5 mM and injected into the thorax of adult females at 18.4 nl per fly using a Nanoject apparatus (Drummond™).

For the measurement of *in vivo* persistence, 9.4 nl of bacterial culture at O.D. 10 were injected into the thorax of female adults (3–4 days old) using a Nanoject apparatus (Drummond™). Persistence of the bacteria was evaluated at 24 and 48 h post-infection by crushing 20 flies in either BHI (*L. innocua*) or LB (*E. carotovora, B. megaterium*) culture medium. Serial dilutions of these extracts were spotted in triplicate on appropriate medium and incubated overnight at 29°C (*E. carotovora*) or 37°C (*L. innocua, B. megaterium*). Colonies were counted from spots containing more than 10 colonies.

Oral infections were performed by feeding adults with an O.D. 200 pellet of *E. carotovora* supplemented with sucrose, as described previously [40].

### Western blot analysis

Western blots were performed with a mouse polyclonal antibody directed against recombinant PGRP-LB (30 kDa) that also recognizes PGRP-SB1. Indeed, a band of about 18 kDa was detected in wild-type fly extracts 6 h after *E. carotovora* injection as well as in unchallenged flies over-expressing *PGRP-SB1* (*daughterless-Gal4, UAS-PGRP-SB1*), but not in extracts from *PGRP-SB* ^Δ5^ mutants (Fig. 3B). A monoclonal anti α-tubulin antibody (Molecular Probes™) was used as a loading control. Samples corresponding to hemolymph from 50 female flies (extracted as described in [8]), were lysed in 2× Laemmli solution. 15 µg of the protein extracts were loaded on a 7.5% SDS-polyacrylamide gel. Following SDS-PAGE, proteins were blotted onto Hybond ECL nitrocellulose membranes (Amersham LIFE Science). The blots were developed using the ECL system (Amersham) and X-ray films.

### Quantitative real-time PCR (RT-qPCR)

For *Dpt*, *Drs*, *PGRP-SB1* and *PGRP-SB2* mRNA quantification from whole animals or guts, RNA was extracted using RNA TRIzol™. For whole animals, 10 flies were used for each sample; for guts, 20 dissected guts from the crop to just above the malpighian tubules were used. cDNAs were synthesized using SuperScript II (Invitrogen) and PCR was performed using dsDNA dye SYBR Green I (Roche Diagnostics). Primer pairs for *Dpt* (sense, 5′-GCTGCGCAATCGCTTCTACT-3′ and antisense, 5′-TGGTGGAGTGGGCTTCATG-3′), *Drs* (sense, 5′-CGTGAGAACCTTTTCCAATATGATG-3′ and antisense, 5′-TCCCAGGACCACCAGCAT-3′), *PGRP-SB1* (sense, 5′-ATGAACACATCAACGGCA-3′ and antisense, 5′-CCGGAAATCCTAGAAGGC-3′), *PGRP-SB2* (sense, 5′-GCTCTCGTTCTATGTGGA-3′ and antisense, 5′-CCCTGAACTTTCTGCG-3′) and *RpL32* (sense, 5′-GACGCTTCAAGGGACAGTATCTG-3′ and antisense, 5′-AAACGCGGTTCTGCATGAG-3′) were used. SYBR Green analysis was performed on a Lightcycler (Roche). The amount of mRNA detected was normalized to control *RpL32* mRNA values. We used normalized data to quantify the relative level of a given mRNA according to cycling threshold analysis (ΔCt), hence data are plotted for example as ΔCt *Dpt*/ΔCt *RpL32*.

### Purification of peptidoglycan and peptidoglycan fragments

Gram-negative and Gram-positive PGN preparations were described in [20]. *E. coli* PGN was purified from the BW25113 Δ*lpp*::Cm^R^ strain that does not express the Braun lipoprotein. Digestion of this material by pure SltY lytic transglycosylase was used to produce mg quantities of TCT and dimer of TCT purified as described previously [29]. Radiolabelled PGN was generated by incorporation of *meso*-[^3^H]DAP in the FB8 *lysA*::*kan* strain grown in M63 minimal medium supplemented with 0.2% glucose and 100 µg.ml^−1^ of lysine, threonine and methionine, as previously described. Muropeptide fragments of PGN were generated by digestion with mutanolysin as described in [29]. Muropeptides were not reduced before incubation with PGRP-SB1 and HPLC separation. In these conditions they are eluted in two peaks (anomers α and β).

Amino acid and amino sugar compositions were determined and quantified with a Hitachi L8800 amino acid analyzer (ScienceTec) after hydrolysis of samples with 6 M HCl for 16 h at 95°C. The structure and purity of isolated PGN fragments and synthesized compounds were further confirmed by MALDI-TOF mass spectrometry.

### Expression of recombinant PGRP-SB1 and assay for *N*-acetylmuramoyl-L-alanine amidase activity

His-tagged PGRP-SB1 was expressed in S2 cells and purified as described previously [32].

The amidase activity of PGRP-SB1 was assayed in a reaction mixture (50-100 µl) containing 50 mM Tris buffer (pH 8), 2 mM ZnSO_4_, pure PGN (100-20 µg) or PGN fragment (2-20 nmoles), and PGRP-SB1 (1-2 µg). After incubation at 37°C for 26 h or 16 h, the reaction was stopped by addition of 2 µl phosphoric or acetic acid and 500 µl HPLC elution buffer: 50 mM sodium phosphate, pH 4.5. To analyze the effects of PGRP-SB1 on the PGN structure in more detail, samples of PGN were first incubated with PGRP-SB1, as described above, for different periods of time, from 20 min to 24 h. Then, reactions were stopped by boiling for 5 min and mixtures were centrifuged. Supernatants were stored for subsequent HPLC analysis of the released soluble peptides (tri, tetra, tetra-tetra…). Pellets of remaining polymeric PGN were treated for 24 h by pure SltY enzyme and the pattern of muropeptides thus generated (TCT, TCT dimer…) was analyzed by HPLC [29]. In all cases, mixtures were injected on a column of Nucleosil 5C_18_ (4.6×150 or 250 mm) (Alltech France) and products were eluted at a flow rate of 0.6 ml.min^−1^ using a gradient of methanol or acetonitrile from 0 to 20% in 40 or 100 min. Peaks were detected either by measurement of the absorbance at 215 nm (and collected for analysis) or by detection of radioactivity (radioactive flow detector, model LB506-C1, Berthold).
